# Predicting Gastric Intestinal Metaplasia in a High-Risk Population

**DOI:** 10.7759/cureus.31502

**Published:** 2022-11-14

**Authors:** Kesiena Akpoigbe, Joan Culpepper-Morgan, Obinna Nwankwo, Alvaro Genao

**Affiliations:** 1 Division of Gastroenterology, Columbia University College of Physicians and Surgeons, Harlem Hospital Center, New York, USA; 2 Division of Internal Medicine, Columbia University College of Physicians and Surgeons, Harlem Hospital Center, New York, USA; 3 Division of Gastroenterology, Columbia University Medical Center Affiliated With Harlem Hospital Center, New York, USA

**Keywords:** gastric intestinal metaplasia black, gastric intestinal metaplasia white, gastric intestinal metaplasia age, gastric intestinal metaplasia hispanics, gastric intestinal metaplasia immigrant, surveillance gastric intestinal metaplasia, screening gastric intestinal metaplasia, racial prevalence, gastric intestinal metaplasia prevalence, gastric intestinal metaplasia

## Abstract

Introduction: Gastric intestinal metaplasia (GIM) is a precancerous lesion. It has a low prevalence rate in the United States. However, GIM is more common among non-White and immigrant populations. Harlem Hospital serves a community that includes predominantly African Americans, Hispanics, and immigrants from West Africa and Spanish-speaking Caribbean countries. This study aims to define the factors predicting GIM in this high-risk group as well as help define screening strategies for vulnerable populations.

Methods: A total of 1351 patients who underwent endoscopic gastroduodenoscopy (EGD) and biopsy in 2018 and 2019 for any indication at Harlem Hospital were included in this study. Gastric biopsy specimens taken during the procedure were assessed for GIM by histopathology. Baseline demographics were collected, including age, sex, and ethnicity. Other information collected included risk factors for GIM such as *Helicobacter pylori* infection, smoking status, and the use of alcohol. Descriptive analysis was done and the Wilcoxon rank sum test and chi-squared test were used to test for associations. Multiple logistic regressions were used to assess the odds of independent factors associated with increased risk of GIM.

Results: Of the 1351 patients reviewed, 106 had GIM for a prevalence of 8.0% (CI: 6.7%-9.6%, p < 0.001). Univariate analysis revealed older patients, males, history of smoking, alcohol, and *H. pylori* infection were significantly associated with GIM. Using multiple logistic regressions and adjusting for underlying risk factors, smoking (OR: 1.61, 95% CI: 1.00-2.570) and *H. pylori* infection (OR: 3.35, 95% CI: 2.18-5.15) continued to be significantly associated with increased risk of GIM; however, alcohol use was not significant after adjusting for other risk factors (OR: 1.10, 95% CI: 0.68-1.78). Hispanic risk for GIM was slightly higher than African Americans (OR: 1.17, 95% CI: 0.74-1.83). The predicted marginal effect of age on the odds of GIM was significant from age 40 and increased exponentially at age 50. By age 70, the odds of GIM were as high as 11% (95% CI: 8.3-13.6).

Conclusion: The prevalence of GIM in our population is significantly higher compared to reported cases in the United States. Age, male gender, *H. pylori* infection, and smoking significantly increase the risk of GIM. Given the high prevalence of GIM in our population, early endoscopic screening would play an important role in evaluating dyspepsia to diagnose GIM with or without *H. pylori* infection. We propose screening all at-risk ethnicities from age 40 years with EGD according to the Sydney System biopsy protocol. We believe this will ultimately decrease the incidence of gastric cancer death in these vulnerable populations of color.

## Introduction

Gastric cancer is the second most common cause of cancer deaths worldwide [1]. It is more common than esophageal cancer [2,3]. It was the 15th most common cancer in the USA in 2018 with a rate of 7.2 per 100,000 new cases of men and women per year and a death rate of 2.9 per 100,000 men and women per year [4,5]. It is observed to be two- to three-fold higher in non-White populations, which include Hispanics, Asians, African Americans (AAs), and Native Americans [6-9]. The annual percentage rise among young Hispanic men in the USA is of particular concern [10,11]. This trend is seen especially in expatriates. The incidence rate for Hispanic males is the highest among non-White populations [9,12,13].

As a precancerous lesion, gastric intestinal metaplasia (GIM) has a low prevalence rate of 4.8% in the United States and especially among Whites [1,4]. However, GIM is more common among non-White populations and maybe more common in the USA than previously thought with crude estimates among Hispanics and AAs as high as 50% compared to 13% for non-Hispanic Whites [6,14,15]. *Helicobacter pylori* is a WHO class I carcinogen like smoking. It is significantly associated with a higher risk of GIM and gastric cancers in racial minorities in the USA [16-19]. It is more common among immigrant populations, AAs, Hispanics, and Asian populations [18,20]. The increased frequency of *H. pylori*, GIM, and gastric cancer in non-White populations may justify the initiation and maintenance of a focused screening program in these communities [21]. This is an area of substantial controversy, especially regarding when to initiate such screening [22].

Harlem Hospital serves one such community that is predominantly AA (65%) and Hispanic (30%). Approximately a third of them are immigrants from West Africa and Spanish-speaking Caribbean countries. This study aims to define the factors predicting GIM in this high-risk group as well as to help define screening strategies for vulnerable populations.

Part of this article was previously presented as a meeting abstract at the 2022 Digestive Disease Week on May 22, 2022, highlighting the risk of GIM in people of color in the USA.

## Materials and methods

Study design and patients

This was a retrospective cohort analysis of Harlem Hospital patients who had endoscopic gastroduodenoscopy (EGD) from January 2018 to December 2019 for any indication. A total of 1351 patients had an EGD procedure done during this time. Random biopsies for histology were taken during the procedures. Histological diagnosis of GIM was extracted from the pathology report. Deidentified data of all patients were obtained from the electronic medical record system. Demographic variables were recorded for each patient. These included age at diagnosis, sex, race, and ethnicity. Other information extracted were risk factors for GIM such as *H. pylori *infection, smoking status, and use of alcohol.

Statistical analysis

Deidentified data obtained from the electronic medical record was sorted, coded, and matched with underlying demographic characteristics and risk information. Analysis of data was contingent on the type of variable. The median, frequencies, and interquartile range (IQR) were used to summarize continuous variables while proportions and percentages were used to describe and summarize categorical data. Where appropriate, the 95% confidence interval (CI) was inferred. Chi-squared statistics and Wilcoxon rank-sum test were used to test for association between age, risk factors, and GIM. Multiple logistic regression was used to assess for independent factors associated with increased risk of GIM. All statistical tests were considered significant at p < 0.05. Analysis was conducted using STATA statistical software version 13 (StataCorp LLC, College Station, TX).

## Results

Of 1351 patients who had EGD between 2018 and 2019, 106 had GIM for a prevalence of 8.0% (CI: 6.7%-9.6%, p < 0.001). This was significantly different from the national average of 4.8% (p < 0.05). Females accounted for 64.5% of the patients in the study. The median age of the study population was 55 years (IQR: 43-65 years) (Table 1).

**Table 1:** Baseline characteristics

Characteristics	N (%)
Age	Median 55 (interquartile range: 43-65)
Sex	
Male	469 (64.5)
Female	852 (35.5)
Race	
Blacks	712 (53.9)
Hispanics	502 (38.0)
Others	107 (8.1)
Risk factors	
Smoking	475 (36.0)
Alcohol	410 (31.0)
*Helicobacter pylori* infection	361 (27.3)
Gastric intestinal metaplasia	
Positive	106 (8.0)
Negative	1215 (92.0)

**Figure 1:**
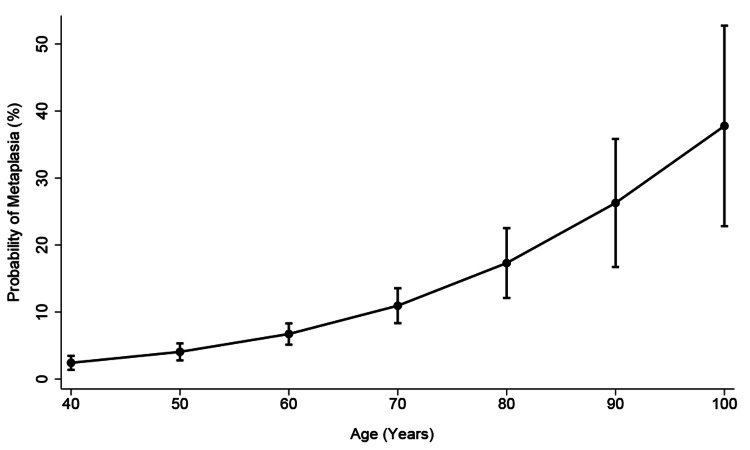
Predicted margins of age and gastric intestinal metaplasia with their 95% confidence intervals

The majority of the study population were AAs (53.9%), followed by Hispanics (38.0%) and others (9.7%). Others included non-Hispanic Whites and Asians. The median age for AAs was 58.1 years, for Hispanics was 50.3 years, and for others was 52.1 years (Table 1). The difference in age between AAs and Hispanics was statistically significant (p < 0.001). About a third of the study population had a smoking history and used alcohol. Of the patients, 27% had *H. pylori* infection. There was a significant association between increasing age and GIM. This relationship started at age 40 and then increased markedly at age 50 as shown in Figure 1.

**Figure 2:**
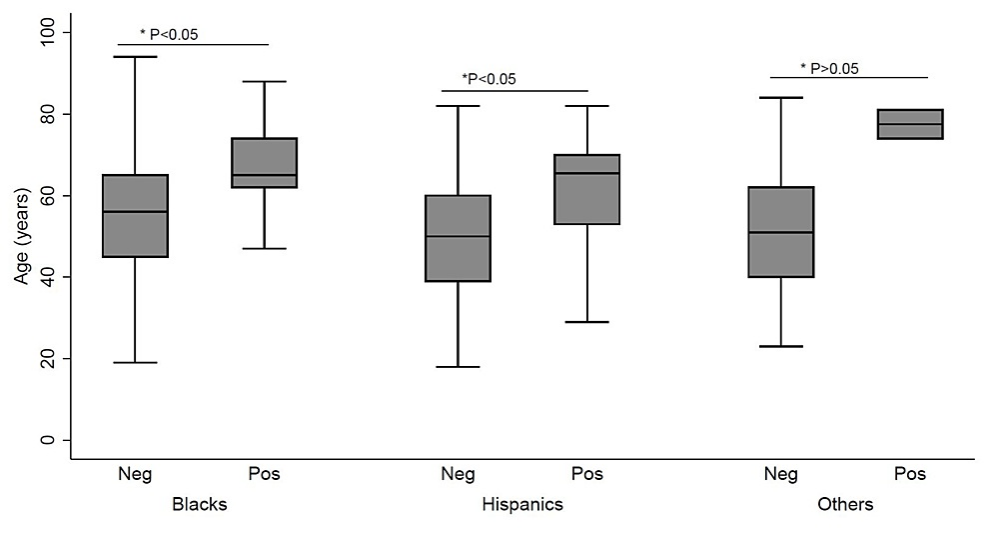
The association between gastric intestinal metaplasia, age, and race Neg, negative gastric intestinal metaplasia; Pos, positive gastric intestinal metaplasia. These are subcategorized under their respective races: Black, Hispanic, and others.

Older Blacks and Hispanics had a significantly increased association with GIM (Figure 2).

Significantly more males (12.4%) than females (5.6%) had a strong association with GIM (p < 0.001). There was no significant association between race and GIM in this overwhelmingly AA and Hispanic cohort. While the rates among AAs and Hispanics were 9.0 and 7.8, respectively, it was still higher as compared to other ethnicities (2.8) (Table 2).

**Table 2:** Prevalence of gastric intestinal metaplasia (GIM)

	N (%) of the population					The proportion of the population with:		
						Positive GIM (95% CI)		Negative GIM (95% CI)
Study population		1351 (100)				8.0 (6.7-9.6)		92.0 (90.3-93.3)
Blacks		712 (53.9)				9.0 (7.1-11.3)		91.0 (88.7-92.9)
Hispanics		502 (38.0)				7.8 (5.7-10.5)		92.2 (89.5-94.3)
Others		107 (8.1)				2.8 (0.9-8.5)		97.2 (91.5-99.1)

The association of GIM with those with a history of alcohol use (10.5%), smoking (11.8%), and *H. pylori* infection (14.4%) was highly significant (p < 0.05) (Table 3).

**Table 3:** Association between gastric intestinal metaplasia and age, race, sex, and risk factors IQR, interquartile range.

	Positive, n (%)	Negative, n (%)	Total	Chi-square (p-value)
Age, median (IQR)	65 (IQR: 56-73)	54 (IQR: 42-65)	-	0.000
Sex				
Female	48 (5.6)	804 (94.4)	852	0.000
Male	58 (12.4)	411 (87.6)	469	
Race				
Black	64 (9.0)	648 (91.0)	712	0.087
Hispanic	39 (7.8)	463 (92.2)	502	
Others	3 (2.8)	104 (97.2)	107	
Risk factors				
Alcohol				
Yes	43 (10.5)	367 (89.5)	410	0.027
No	63 (6.9)	848 (93.1)	911	
Smoking				
Yes	56 (11.8)	419 (88.2)	475	0.000
No	50 (5.9)	769 (94.1)	846	
*H. pylori* infection				
Yes	52 (14.4)	309 (85.6)	361	0.000
No	54 (5.6)	906 (94.4)	960	

Adjusting for underlying risk factors, i.e., smoking (OR: 1.61, 95% CI: 1.00-2.57) and *H. pylori* infection (OR: 3.35, 95% CI: 2.18-5.15), significantly increases the odds of GIM. However, alcohol use did not reach statistical significance after adjusting for other risk factors (OR: 1.10, 95% CI: 0.68-1.78). The odds of Hispanics having GIM was slightly higher than AAs (OR 1.17, 95% CI: 0.74-1.83) after adjusting for other risk factors. Although this was not statistically significant, it is a trend worth noting given the size of their population as compared to AAs. Increasing age (OR: 1.05, 95% CI: 1.04-1.07) and male sex (OR: 1.91, 95% CI: 1.25-2.93), respectively, increased the odds for GIM (Table 4).

**Table 4:** Factors predicting gastric intestinal metaplasia

	Crude (OR, CI)	P-value	Adjusted (OR, CI)	P-value	
Age	1.05 (1.03-1.07)	0.000	1.05 (1.04-1.07)	0.000	
Sex					
Female	Reference		Reference		
Male	2.36 (1.58-3.52)	0.000	1.91 (1.25-2.93)	0.003	
Race					
Black	Reference		Reference		
Hispanic	0.85 (0.56-1.29)	0.453	1.17 (0.74-1.83)	0.501	
Others	0.29 (0.09-0.94)	0.040	0.35 (0.11-1.17)	0.087	
Risk factors					
Alcohol					
No	Reference		Reference		
Yes	1.58 (1.05-2.37)	0.028	1.10 (0.68-1.78)	0.704	
Smoking					
No	Reference		Reference		
Yes	2.13 (1.43-3.17)	0.000	1.61 (1.00-2.57)	0.049	
*H. pylori* infection					
No	Reference		Reference		
Yes	2.58 (1.89-4.22)	0.000	3.35 (2.18-5.15)	0.000	

The predicted marginal effect of age on the odds of GIM for those less than age 40 was 2% (CI: 1.3%-3.5%, p < 0.05). By age 50, the predicted marginal effect of age on the odds of GIM increased exponentially to 4% (CI: 2.8%-5.3%, p < 0.05), as shown in Figure 1 and Table 5. By age 70, the predicted marginal effect of age on the odds of GIM was shown to be 11% (Figure 1 and Table 5).

**Table 5:** Predictive margins of age and gastric intestinal metaplasia

Age (years)	Margin	Standard error	Z	P > Z	95% CI
40	0.024	0.005	4.51	0.000	0.014-0.035
50	0.041	0.006	6.29	0.000	0.028-0.053
60	0.067	0.008	8.34	0.000	0.052-0.083
70	0.110	0.013	8.19	0.000	0.083-0.136
80	0.173	0.027	6.52	0.000	0.121-0.225
90	0.263	0.049	5.40	0.000	0.167-0.358
100	0.378	0.076	4.96	0.000	0.228-0.527

## Discussion

The prevalence of GIM in our study population was 8.0%. This was about twice the national rate of 4.8%. It was much higher still when it was subcategorized on ethnicity [9,12,13]. AAs had a prevalence rate of 9.0% and Hispanics had a rate of 7.8%. Other studies have reported a prevalence of GIM in Hispanics of 12.2% and AAs of 9.7%, which is even higher [1,15,23,24]. A prospective study that included asymptomatic patients from a Veterans Affairs hospital in Houston interestingly found a prevalence of 29.5% among Hispanics, 25.5% among AAs, and 13.7% among non-Hispanic Whites. The reason for their much higher prevalence was likely due to the older age (five years) and strong male predominance (90%) of their cohort.

We were likely unable to detect a difference in prevalence between AAs and Hispanics due to the similar rate of increased risk in these populations vs. Whites. While it appears that the prevalence rate of GIM was higher in AAs in our study, after adjusting for other risk factors such as smoking, alcohol, and *H. pylori* infection, the odds of having GIM in Hispanics was about 17% higher than in AAs. This further buttressed the trend observed in Hispanics especially among expatriates as Harlem Hospital serves predominantly an immigrant population. It is also important to note that our AA cohort was about seven years significantly older than our Hispanic cohort. This also may have driven the prevalence rate upward.

Age was a significant factor associated with GIM in this study population. The median age for GIM was 65 years (IQR: 56-73). Other studies have reported average ages of GIM from 60 years to over 70 years [15,23,24]. While the median age for the study population was 55 years, the predicted marginal effect of age on the odds of GIM became significant from age 40 and markedly increased after age 50. This implies that the higher number of GIM seen in the older population might have actually started at a younger age and makes it more important for any form of screening or surveillance program to be considered at the younger age of 40 than 50. This effect of age holds true for both Hispanics and AAs.

Several of the risk factors in this study were associated with increased odds of GIM. *H. pylori*, a carcinogen, is strongly associated with GIM [15,25-30]. There was a higher percentage of GIM with *H. pylori *infection (14.4% vs. 5.6%) in this study. It significantly increased the odds of GIM in this population by 3.35. It was the most significant risk factor for GIM in this population. Nguyen et al. also found that *H. pylori* is the most significant risk factor for GIM in Hispanics, AAs, and non-Hispanic Whites. It was said to be associated with over five-fold increase in the risk in non-Hispanic Whites such that testing for *H. pylori* could be used as a surrogate for the presence of GIM. However, only 34% of the risk for GIM could be attributed to *H. pylori* in AAs and Hispanics. Therefore, other unknown factors may have accounted for the increased risk in these racial and ethnic groups. Given that our population is an immigrant population that comes from regions where *H. pylori* is endemic, it would be beneficial to initiate early screening for detection and treatment as studies have shown it to be more cost-effective in Hispanics and AAs in reducing gastric cancer [31-34].

Smoking also has a strong association with GIM. It increases the odds by 61%. Smoking like *H. pylori* has been associated with GIM and also gastric cancer [35,36]. While this would not be surprising, it lays further emphasis on the role that both *H. pylori* and smoking could play in tandem for initiating GIM and gastric cancer. Of note, alcohol was observed to have an association with GIM but the odds of causing it was not significant after adjusting for *H. pylori* and smoking. This is in keeping with other studies, which show it does not have any significant effect on causing GIM [37].

Most of our study population was female. They were about two-thirds of all persons who had an EGD between 2018 and 2019. The prevalence of GIM in females was 5.6%, i.e., about half the prevalence in males (12.4%). This association is in keeping with males having higher odds of GIM (1.19 (1.25-2.93)) than females. In addition, males have been noted to have a higher rate of GIM in other studies [15,18,26,38,39].

Current American College of Gastroenterology (ACG) dyspepsia guidelines recommend that we test and treat *H. pylori* for patients under the age of 60 with EGD reserved for those 60 and older. We feel this would not allow for the diagnosis and surveillance of patients with high-risk lesions such as GIM, many of whom have already lost their *H. pylori*. The Correa cascade suggests that GIM is an irreversible lesion that may progress to dysplasia in over 3.2 years [40]. It is unclear if *H. pylori* eradication alone is enough to completely interrupt this cascade. In addition, many of these patients may become reinfected as they return to their country of origin periodically. The U.S. Department of Homeland Security (DHS) Office of Immigration Statistics (OIS) estimated in 2016 the average re-entry per nonimmigrant per year was 1.8 times per person and could be as high as 4.4 times per person for Mexicans [41].

There are limitations to this study. Categorizing all Blacks in the study as AAs could have missed the contributions that African immigrants may have made independently since they are likely to have come from areas endemic to *H. pylori* infection [42-47]. Also lumping Hispanics into one group could have masked the different effects Caribbean Hispanics might have had from Mexican American Hispanics, South American Hispanics, etc. Another limitation is that we did not stratify our result based on the indication for EGD. This may have provided some additional insight into the selection of patients for screening by EGD. The number of pack years of smoking would also have been useful as smoking as a carcinogen has a dose response [48-51]. Some studies have even found that ever smokers were as much at risk for GIM as current smokers [52]. Lastly, the site of GIM was not recorded as it would have been helpful to note the predominant site in the stomach with GIM. Antral GIM has been found in other studies to be the most common site [53-55]. It is also the most common site for gastric cancer [56,57].

The strengths of this study are that it was a real-world practice study. Biopsies were not done according to the Sydney GIM screening protocol but were done simply to diagnose *H. pylori*. Therefore, it is likely that we underestimated the prevalence of GIM in all groups. Also, our cohort was younger and included females. As a result, we were able to detect the early rise in the probability of GIM at age 40 with a significantly narrow confidence interval (Figure 2). Thus, we recommend that screening for this precancerous lesion should begin at age 40.

## Conclusions

The prevalence of GIM in our population is significantly higher compared to the reported prevalence in the United States. Age, male gender, *H. pylori *infection, and smoking significantly increase the risk of GIM in AAs and Hispanics. Given the high prevalence of GIM in our institution, early endoscopic screening could play an important role in evaluating dyspepsia to diagnose GIM with or without *H. pylori* infection, not only to prevent this precancerous lesion from developing but to identify those with irreversible GIM with dysplasia that may need surveillance. Thus, we propose screening all at-risk ethnicities from age 40 with EGD according to the Sydney System biopsy protocol. We believe EGD screening for GIM will ultimately decrease the incidence of gastric cancer death in these vulnerable populations of color.
